# A qualitative study on aspects of consent for genomic research in communities with low literacy

**DOI:** 10.1186/s12910-020-00488-0

**Published:** 2020-06-12

**Authors:** Daima Bukini, Columba Mbekenga, Siana Nkya, Lisa Purvis, Sheryl McCurdy, Michael Parker, Julie Makani

**Affiliations:** 1 Sickle Cell Programme, Department of Haematology and Blood Transfusion, Muhimbili University of Health and Allied Sciences, UN Road, Upanga, Block 9, Dar es Salaam, Tanzania; 2 School of Nursing and Midwifery, Aga Khan University, Dar es Salaam, Tanzania; 3 The Dartmouth Institute for Health Policy & Clinical Practice, Dartmouth-Hitchcock Medical Centre, Hanover, NH USA; 4 University of Texas Health Science Centre at Houston, School of Public Health, Houston, TX USA; 5 Welcome Centre for Ethics and Humanities, University of Oxford, Oxford, UK

**Keywords:** Informed consent, Comprehension, Low literacy, Genomic studies

## Abstract

**Background:**

Low literacy of study participants in Sub - Saharan Africa has been associated with poor comprehension during the consenting process in research participation. The concerns in comprehension are far greater when consenting to participate in genomic studies due to the complexity of the science involved. While efforts are made to explore possibilities of applying genomic technologies in diseases prevalent in Sub Saharan Africa, we ought to develop methods to improve participants’ comprehension for genomic studies. The purpose of this study was to understand different approaches that can be used to seek consent from individuals with low literacy in Sub-Saharan African countries in genomic research to improve comprehension.

**Methods:**

Using qualitative study design, we conducted focus-group discussions, in-depth interviews and participant observations as data collection methods. This study was embedded in a hospital based genomic study on Sickle Cell Disease at Muhimbili National Hospital in Tanzania. Thematic content analysis was used to analyse the transcripts and field notes.

**Results:**

Findings from this study show that literacy level has little influence on understanding the research details. According to the participants of this study, the methods used to provide information, the language, and time spent with the study participants were the key factors influencing understanding. The availability of group sessions held before individual consent to allow for a detailed questions and answers format was agreed to be the best method to facilitate the comprehension.

**Conclusion:**

The quality of the consenting process of participants will be influence by a number of factors. The type of research consented for, where the research will be implemented and who are the potential study participants are amongst the factors that need to be assessed during the consenting. Measures to improve participants’ comprehension need to be developed when consenting participants with low literacy level in genomic studies.

## Background

One of the requirements for conducting ethical research with competent adult participants is obtaining a valid informed consent [1–3]. Studies have suggested that designing and obtaining consent for genomic studies when implemented in Sub-Saharan Africa (SSA) is challenging to the researchers and research participants [4–8]. Often low literacy of the participants has been associated with poor comprehension during the consenting process [9, 10]. The challenges are linked to difficulties in providing information in a comprehensible manner to the participants considering the complexity of the science involved [11, 12]. The process of interpretations and translations of key concepts into accessible terms for the participants can be challenging to achieve for researchers and field workers [6, 13]. This is particularly problematic in populations where the science requires translations into local languages [2, 14–16]. Lack of training in genetics for clinicians and the shortage of genetic counsellors in the SSA may have increased the difficulty in communicating the information to the research participants [17]. Often in this context, a well-written and signed consent by a participant does not guarantee understanding. In contributing to that discussion, we designed an exploratory study aiming to understand research participants’ perspectives and experiences when consented in a genomic study implemented in SSA. We were particularly interested to learn how participants’ literacy level may have had an influence on their understanding the details of the research into which they have enrolled.

## Methods

### Sampling and recruitment

We recruited research participants enrolled in an urban hospital-based study aiming to understand genetic determinants of clinical heterogeneity of sickle cell disease (SCD) in Tanzania [18, 19]. At the time of recruitment, the study consisted of 3751 individuals with SCD, 1779 were between 5 and 17 years of age with almost a 50/50 gender distribution [20]. Participants for the study reported here, were recruited through adult and paediatric clinics at Muhimbili National Hospital. Purposive sampling was used to select individuals willing to provide detailed information about their understanding and experiences of being enrolled in the study. In total forty-three (*n* = 43) participants were recruited into our study. We defined literacy level in relation to the number of years spent in school as per the education system in Tanzania; (1) Primary - basic level of education, also included here were participants with no formal education (*n* = 23); (2) Secondary level - intermediate level (*n* = 16); and (3) Tertiary level - high level of education (*n* = 4).

### Data collection methods

Data was collected through focus-group discussions (FGDs), in-depth interviews (IDIs) and participant observations (POs).

### Focus group discussions (FGDs)

Five FGDs were conducted with six participants in each of the groups. The composition of the FGDs took into consideration age, level of education, and gender. The FGD guide was designed to facilitate a semi-structured discussion around participants’ experiences, challenges and possible ways to improve the consenting process. In total, 30 individuals participated in the five FGDs. Participants included were adult SCD patients enrolled in the study. Details of the participants’ descriptions are provided in Table 1.

**Table 1:** Focus Group Discussions Participants

Description	Participants (*N* = 30)
Male n *(%)*	7 (23)
Age *mean (min, max)*	26 (18, 40)
Education *n (%)*	
No formal education	5 (17)
Primary education- basic level of education	9 (30)
Secondary education- intermediate	12 (40)
Tertiary education- high level of education	4 (13)

### Participant observations and in depth interviews

In addition to the focus groups, we observed the consent process of six new participants who were enrolled in the study during the period of our research. The content of the observation checklist was developed based on the consent form used by the researchers. For instance, we observed how the researcher communicated the objectives and methods of the study to the participants as described in the informed consent. After observing the consenting process, we interviewed those participants (IDIs) to gather their perspectives on the consenting process. In total, 13 IDIs were conducted, out of which seven (7) IDIs were conducted with research participants who were already enrolled in the study. Amongst the participants, nine were parents of children who have been enrolled in the SCD study. Participants were aged 24 to 43 years old, amongst which two (2) were men and eleven (11) women. Since most of the children were brought to the clinic by their mothers, we did end up interviewing more women than men in the study. Details of the participants’ descriptions are provided in Table 2.

**Table 2:** In Depth Interviews participants

Male n *(%)*	2 (13)
Age *mean (min, max)*	33 (24, 43)
Education *n (%)*	
No formal education	3 (13)
Primary	6 (35)
Secondary	4 (23.5)
Tertiary	0 (0)

### Data analysis

We analysed the transcripts and field notes using thematic content analysis adapted from another qualitative study implemented in Tanzania, Ghana and Cameroon [21]. The first author, (DB), collected all the information for IDIs and FGDs as well as recording participants’ observation checklist. The interviews were all done in Swahili and then translated to English after the analysis stage. After the initial coding by DB, co-authors CM and SM completed quality checks by reviewing a selection of the data. The themes were developed inductively, based on the emerging codes. Sorting and coding were continued until no new subcategories could be identified from the data. Data from all three methods were analysed together to check for similarities or differences. The emergent themes are results of the analysis of all methods: IDIs, FGDs and POs.

## Results

Three main themes were identified from our analysis of the data from FGDs, IDIs and POs: 1) Relationship between low levels of literacy and the achievement of informed consent; (2) Factors contributing to poor understanding included: inadequate information provided to the participants, age at enrolment, and time spent by the participants; (3) Possible ways to improve understanding as suggested by participants; opportunities to ask questions and group information sessions followed by individual consenting.

### The relationship between low levels of literacy and the achievement of informed consent

Participants who reported having no formal education and those categorized as having low literacy felt that the possibility of gaining a good understanding of the research was not influenced by having low literacy level. This perception was consistent during the IDIs, FGDs and POs. The following quotations illustrate this.

A response from a mother (IDI 3) who doesn’t know how to read or write was, “*Research in Sickle Cell Disease are [is] done to understand the cure and find solutions on this problem*” IDI 3, interview with a mother of a child with SCD enrolled in the genomic study

Another example was also from a mother (IDI 1) who has not received formal education, she explained, “*Research is an investigation to understand about a certain disease problem in-depth or look for a cure of a certain disease*”” IDI 1, interview with a mother of a child with SCD enrolled in the genomic study

In one of the FGDs, participants were asked whether the information provided by researchers was easy or difficult to understand. One FGD member began,

“*Yes, it is easy to understand*, [A probe was followed after his response, *Is it easy for you because you are a college graduate*? ] Another participant in the group with no formal education replied, “*It is all about the individual person and has nothing to do with the level of education*” focus group discussion 2 with adult SCD patients enrolled in the genomic study

A participant in another FGDs claimed, *“I am not concerned by the type of information provided, it is not a problem for me to understand, we need to be educated more on the research and understanding will not be a problem*” focus group discussion 3 with adult SCD patients enrolled in the genomic study

A few participants believed that education plays a major role to be able to understand the information.

One noted, “*It is true that for someone to understand this (research) information, it will depend on the individual person, but also the level of education they possess*” IDI participant 5, 43 year-old men with SCD who was newly enrolling in the genomic study

### Factors contributing to poor comprehension of the research

#### Adequacy of the information provided to the participants

The majority of participants for both the IDIs and FGDs felt that research information was not adequately provided. Participants describe the quality of the interactions,

“*You need more time to understand the information and if you are not keen enough you will not be able to understand, time given was so short and answers were given in a rush [ed] way*” IDI participant 9, mother of a child with SCD enrolled in the genomic study

A FGDs respondent clarified, “*The truth is we are not getting enough information from the doctors [researchers] and education is not being provided to our satisfaction [on the research], only the progress of your disease, but nothing more...that is at least my personal view*” Focus group discussion 1 with adult SCD patients enrolled in the genomic study

One explanation the participants gave for why they had received inadequate information was an increase in patient load in the clinics, *“I do not think we are now getting information as it used to be, as time goes by the number of clinic visits decreased and the time to stay with a doctor also has decreased”* Focus group discussion 2 with adult SCD patients enrolled in the genomic study

In contrast to the other study participants, however, one woman noted, “*I have been provided with detailed information about the research, they have even come to my house to do research and the experience at home was quite good*” IDI 7, interview with a mother of a child with SCD enrolled in the genomic study.

#### Age at enrollment

Age at enrolment was considered to be a factor by some of the participants for not recalling the information provided during the consenting process. Since participants were recruited from a cohort study, this was only relevant for those who were enrolled in the study by their parents or caretakers when they were young. As commented by one participant in the FGD,

“*Time has passed since the last time we were enrolled, I cannot recall if it was difficult or easy to understand, maybe there is a need to be given the information again*” focus group discussions 5 with adult SCD patients enrolled in the genomic study

Another participant, who was about 16 when enrolled [he was 19 at the time of the interview]*,* recalled: “*I remember my father signing this form [indicating the consent forms], but I was there to witness. I was about 16 years old. I remember my father looking satisfied with the information provided by the doctor. I do not recall being given the forms to go back home and read them*” IDI participant 11, 19 year-old boy with SCD enrolled in the SCD genomic study

#### Time the researcher spent with the study participants

Participants felt that time spent with the researcher was not enough for detailed conversation. They were concerned that the time limitations do not allow them to ask questions.

“*Maybe it's contributed [time spent] to the increase in number of people attending clinics these days, there is no time to listen to the patients one by one, and sometimes the environment is not that comfortable to start asking questions* [Another participant in the same FGD clarified], *It is like this* [commanding people to listen to what she was about to say], *before you even come out, another patient is already at the door waiting for you to come out or sometimes standing inside the room waiting, you cannot ask anything*” focus group discussions 4 with adult SCD patients enrolled in the grnomic study

### Possible way to improve understanding during the consenting process

#### Opportunity to ask questions enhances participants’ comprehension

Participants who had opportunities to ask questions, recall how it helped them to understand the details of the research.

“*We were given opportunities to ask questions, doctors I recall responded very well and we were satisfied, I am now confident that I have a fairly good understanding of the disease*” IDI 6, interview with a mother of a child with SCD enrolled in the genomic study

“*For those who are upfront they can ask questions to the doctors but the doctors they just do not start to provide explanation, for example, the tests we are doing at the clinic*, [it is not] *until you ask what are they for and what do these results mean* [that you are given an explanation]” focus group discussions 3 with adult SCD patients enrolled in the genomic study

#### Group information sessions followed by individual consenting

Participants preferred group informational sessions followed up by individual meetings for signing the consent forms. For example, a woman in one FGD noted she preferred a group session,

“*[That would be] like this [indicating the group discussions], in the presence of doctors … because it can be very difficult to ask questions through the one to one method with a doctor … some people feel ashamed to ask questions but in the group discussions you do not feel that*” focus group discussions 4 with adult SCD patients enrolled in the genomic study

Another participant recalled, “*We were provided the information in groups of five people and I think it helped to remember, although we both signed individually*” focus group discussions 1 with adult SCD patients enrolled in the genomic study

In addition to preferring a two-stage general informational session and an individual consent signing process, participants preferred face-to-face encounters to getting information from a video, this comment was raised when participants were asked about having videos in the clinics to explain the study,

“*I am sceptical with the video method of providing information, I am not sure of it, it depends on the research type, you just cannot show everything*” focus group discussions 1 with adult SCD patients enrolled in the genomic study

## Discussion

When we began this research, we expected low literacy to be one of the factors hindering comprehension at the time of consent to participate in genomic research. However, our findings and the analysis of our data have shown that participants believe that understanding of the concepts in genomic research were dependent more on the methods used to communicate the information [22]. These results were similar to those of a study conducted in Gambia and Bangladesh [10, 23, 24] which indicated that ways in which information is provided is the most important component influencing success of the consenting process.

Limitation of time was also a contributing factor for researchers not to adequately provide information. Group interactions were thought by participants as very helpful to assist them recall the information and provided them with opportunities to ask any questions they have to the doctors as compared to the one-on-one session. In addition to that, the group sessions before one-to-one sessions will help minimize time the researchers will spend with individual participant to explain the research.

Although participants acknowledged that asking questions can enhance comprehensions, only few participants felt comfortable asking questions about the research. Fear of being seen as ignorant, trusting that doctors know it all and the power imbalance between the researcher and the subject especially when the doctor is also the one recruiting subjects limit participants willingness to ask questions [14, 25–27]. One way of addressing this is might be by having the research team different from the clinical team.

Participants who enrolled in the study when they were young could not recall the information provided during the consenting process. Some vaguely recall their parents consenting on their behalf. This explains the importance of re-consenting study participants after a certain time period depending on the study’s risk level and duration. The re-consenting process emphasized is not just asking participants if they are willing to continue being part of the study, but rather refreshing on the purpose of the study, procedures involved, risks as well as benefits of the research.

In our interviews and focus groups, in addition to highlighting challenges, participants suggested alternate research presentation methods that could be used to improve the effectiveness and accessibility of consent processes. Suggestions made included the use of practical examples and simple terminologies that can be easily understood by the participants [28, 29]. Participants in this study felt that the quality of the information provided was compromised by the limited time doctors have to spend on the consenting process. It was felt that providing information in groups through seminars, in the presence of nurses or doctors as moderators, would help to save time required during the one-on-one session. In-group sessions, the researcher may try to ensure all the general components of the informed consent such as purpose, risks, procedures, benefits and dissemination plans are explained in detail [30]. By using a group session approach, researchers who may also be physicians, will not be required to spend a lot of time explaining research to individual participants. This particular arrangement will not substitute individual autonomy because the aim will be to empower the subjects to make the right informed decision, after understanding the study details. This is also supported by a study done on tailoring consent to context [23], that in some settings individual consent may benefit from being preceded with providing information in groups. However, there are studies that suggested that there were no overall benefits of providing information in groups before individual consent [31].

Since this study was done in a hospital setting, participants sometimes seem to confuse research methods and clinical practices. At times it was difficult to tell whether the experiences they were referring to, were research or clinical experiences. We also acknowledge that the experiences shared by participants in this study do not seem to be specific to genomic research, and, therefore, the findings can be broadly interpreted to other non-genomic research.

## Conclusion

This study afforded us with an opportunity to reflect on what kind of information is being provided to the participants and how. Our findings suggest that the reason why comprehension is difficult is not solely due to low literacy of the participants but also, perhaps mostly, down to how the information is being provided. Researchers conducting research in the low-literacy settings have to invest in understanding the best methods to use to provide the research information in a much more accessible and comprehensible manner. Participants’ low literacy levels have always been used as a scapegoat for poor comprehension, but by placing the blame on participants we have failed to acknowledge our failure to consider the context and adequately provide clear explanations of our research. Our recommendation to researchers is to plan the consenting process ahead of time and plan for a re-consenting phase for studies that have a long time span, to factor in the context of where the research will be carried out, and to better understand the needs of the target population to and align their needs to efforts to fully inform the consenting process. However, we do acknowledge that participants recruited in this study may have different consenting experiences due to several factors at the time of enrolment. Those factors may have largely contributed to their own perspectives on understanding of the research.

## Data Availability

The datasets used and analysed during the current study are available from the corresponding author on reasonable request.

## Abbreviations

SCD: Sickle Cell Disease
SSA: Sub Saharan Africa
FGDs: Focus Group Discussions
IDIs: In- Depth Interviews
POs: Participant Observations
